# The efficacy of modified bloodless del Nido cardioplegia in isolated aortic valve replacement

**DOI:** 10.1371/journal.pone.0333083

**Published:** 2025-09-29

**Authors:** Bongyeon Sohn, Jae Suk Yoo, Dong Jin Kim, Heemoon Lee

**Affiliations:** 1 Department of Thoracic and Cardiovascular Surgery, Bucheon Sejong Hospital, Bucheon, Gyeonggi-do, Republic of Korea; 2 Department of Thoracic and Cardiovascular Surgery, Asan Medical Center, Seoul, Republic of Korea; Royal Holloway University of London, UNITED KINGDOM OF GREAT BRITAIN AND NORTHERN IRELAND

## Abstract

This study aimed to evaluate the safety and efficacy of a modified, bloodless Del Nido (DN) cardioplegia solution in patients undergoing isolated aortic valve replacement (AVR). A total of 370 patients who underwent isolated AVR between 2015 and 2022 were retrospectively analyzed. Patients were categorized into two groups based on the cardioplegia solution used: the bloodless DN group (N = 180) and the histidine-tryptophan-ketoglutarate (HTK) group (N = 190). To reduce selection bias and adjust for baseline differences, inverse probability of treatment weighting analysis was performed. There was no significant difference in in-hospital mortality between the two groups (HTK vs. DN: 1.2% vs. 0.9%, P = 0.554). However, the rate of spontaneous sinus rhythm restoration without the need for defibrillation following aortic cross-clamp release was significantly higher in the DN group (40.0% vs. 75.2%, P < 0.001). Additionally, the initial postoperative lactate level (3.0 ± 2.6 mmol/L vs. 2.2 ± 1.4 mmol/L, P = 0.002), and the incidence of low cardiac output syndrome (9.4% vs. 1.7%, P < 0.001) were significantly lower in the DN group compared to the HTK group. Other postoperative morbidities did not differ significantly between the groups. The modified bloodless Del Nido cardioplegia demonstrated favorable myocardial protection and early clinical outcomes compared to HTK solution in patients undergoing isolated AVR. These findings suggest that the bloodless Del Nido technique may be a viable alternative, although further validation in larger, prospective studies is warranted.

## Introduction

Myocardial protection is an essential element for successful cardiac surgery since left ventricular failure resulting from ischemic myocardial damage is a major cause of mortality and morbidity following cardiac surgery [1]. Myocardial protection in aortic valve replacement (AVR) is particularly challenging because left ventricular hypertrophy and wall tension in aortic valve disease increase myocardial oxygen demand [2,3]. Although several myocardial protection strategies with different cardioplegia have been suggested, the optimal myocardial protection strategy is still controversial in aortic valve surgery [4–7].

The histidine-tryptophan-ketoglutarate (HTK) cardioplegia is a single-dose cardioplegia, offers over two hours of myocardial protection, and provides favorable clinical outcomes in various cardiac surgeries including aortic valve surgery [4,5,8]. However, several drawbacks of HTK solution including, hemodilution, disturb blood homeostasis, electrolyte imbalances have been reported [9,10].

The Del Nido (DN) cardioplegia, introduced in the 1990s as a singular cardiac protection solution primarily for congenital and pediatric heart surgeries, has exhibited a reliable duration of myocardial safeguarding exceeding 60 minutes [11–13]. Its application has extended to diverse adult cardiac surgeries with consistently positive outcomes [14–17]. It’s noteworthy that with the expiration of the patent for DN cardioplegia, there has been a proliferation of modified versions, and research endeavors have been reported to explore and optimize its formulation [18,19].

In our institution, DN was firstly adapted in congenital heart surgery. The original formulation requires mixing with blood, which adds complexity to the circuit setup. In certain cases, constructing the mixing circuit may be technically challenging, unavailable due to equipment limitations, or impractical if additional blood withdrawal is not feasible. This is particularly relevant in pediatric patients, who are more sensitive to changes in blood volume. Under these circumstances, we began using a bloodless version of the del Nido solution since early 2000s. Favorable outcomes of modified ‘bloodless’ DN formula for several years in congenital cardiac surgery have broadened usage of the modified version of DN cardioplegia into adult cardiac surgery in 2014. The aim of our study is to assess the effectiveness of our modified ‘bloodless’ DN cardioplegia in AVR, with a comparative analysis against HTK cardioplegia.

## Materials and methods

### Study design

In a retrospective analysis, we examined the records of 370 patients who underwent isolated AVR at our institution from September 2015 to March 2022. These patients were categorized into two groups: HTK cardioplegia (N = 190) and DN cardioplegia (N = 180). The study protocol was approved by the Institutional Review Board of Bucheon Sejong Hospital, which waived the requirement for informed patient consent (IRB No. BSH 2023-04-001; approval date: April 19, 2023). Data used in this study were accessed for research purposes between 17/05/2023 and 16/05/2024. All procedures were conducted in accordance with relevant guidelines and regulations.

### Surgical techniques and myocardial protection strategies

The patients in our study underwent AVR procedures through either a full median sternotomy or a minimally invasive approach, which included right mini-thoracotomy, right anterior thoracotomy, and partial sternotomy. The surgical operations were conducted under mild hypothermia. For myocardial protection, antegrade perfusion of a cardioplegic solution was employed. In patients without aortic regurgitation, antegrade cardioplegia was infused via aortic root cannula after aortic cross-clamping (ACC). In patients with significant aortic regurgitation, cardioplegic solution was administered directly to the coronary arteries after aortotomy following ACC. The aortic valve replacement was performed with the standard fashion. A biological, a sutureless, or a mechanical prosthesis was implanted based on age and choice of patient regarding to the clinical situations.

### HTK cardioplegia

HTK solution was utilized in its commercially prepared, original form. The composition of the HTK solution is detailed in Table 1. The initial dose of the HTK solution was standardized at 2,000 mL. To achieve myocardial protection, the cardioplegia was administered antegradely into the aortic root, maintaining a 4 °C infusion temperature. In instances where the ACC time surpassed 120–180 minutes, an additional dose, equivalent to half of the initial dose, was administered to sustain effective cardioplegic protection.

**Table 1:** Composition of cardioplegias.

HTK		Bloodless DN	
Water for injection	1L	Plasma-Lyte A base solution	1L
NaCl	0.8766g	Mannitol 15%	21.7mL
KCl	0.6710g	Potassium chloride (2mEq/mL)	13mL
MgCL2.6H2O	0.8132g	Sodium bicarbonate 8.4%	13mL
Mannitol	5.4651g	Magnesium sulfate 50%	4mL
Histidine	27.9289g	Lidocaine 1%	13mL
Tryptophan	0.4085g	20% Dextrose solution	10mL
Histidine.HCl.H2O	3.7733g		
CaCl2.2H2O	0.0022g		
2-Ketoglutarate-H-K	0.1842g		

HTK, Histidine-tryptophan-ketoglutarate; DN, Del Nido.

### The modified bloodless DN cardioplegia

The DN cardioplegia was carefully prepared by perfusionists, containing the same components as the original formulation, as outlined in Table 1. The formula contains components nearly consistent with those of the original del Nido solution, except that 15% mannitol was used instead of 20%. Unlike the conventional protocol, no blood components were mixed during administration, rendering it a fully crystalloid solution. Instead, 10 mL of 20% Dextrose solution was added as an energy substrate. The initial dose was standardized at 600 mL per body surface area (BSA) and delivered via antegrade perfusion into the aortic root at an infusion temperature of 4 °C. In cases where the ACC time exceeded 90 minutes, an additional dose—equivalent to half of the initial volume—was administered using the same technique to maintain effective myocardial protection.

### Statistical analysis

Descriptive statistics for the entire study population were computed, with categorical variables presented as numbers and percentages, and continuous variables as means and standard deviations. To assess inter-group differences, the t-test (or the Mann–Whitney test when the normality assumption was in doubt) and Chi-square test (or Fisher’s exact test when the expected cell frequency was < 5) were employed for continuous and categorical variables, respectively. An inverse probability of treatment weighting (IPTW)-adjusted analysis was performed to balance the distribution of baseline risk factors between HTK and DN groups. The propensity score was derived through multiple logistic regression, considering preoperative baseline characteristics and operative parameters. They included chronic obstructive pulmonary disease, New York Heart Association (NYHA) class 3–4, Atrial fibrillation, previous open heart surgery, left ventricular ejection fraction, aortic stenosis, aortic steno-regurgitation, infective endocarditis, cardiopulmonary bypass time and aortic cross clamp time. Weights for the DN group were the inverse of the PS, and those for the HTK group were the inverse of 1-PS. Stabilized weights were used to reduce variability in the IPTW model. The love plot and the density plots before and after IPTW were included in S1 and S2 Figs, respectively. We also analyzed PS matching as an added robust analysis result. A total of 104 patients from the DN group were matched to 104 patients from the HTK group using nearest-neighbor matching without replacement and a matching tolerance (caliper) of 0.2. The Cox proportional hazards model analysis was employed to estimate the treatment effect of the two groups on long-term outcomes. The hazard ratios (HRs) of late clinical outcomes between the two groups were compared based on original unmatched data, IPTW models, and matched data. There was no missingness for data in our models and no imputation was performed for missing data. Statistical significance was set at P < 0.05. Statistical analysis was carried out using R 4.3.1 (R Foundation for Statistical Computing, Vienna, Austria).

## Results

### Baseline characteristics

Patients of the DN group had a higher prevalence of New York Heart Association functional classification 3 or 4 (HTK vs. DN; 10.5% vs. 25.0%, P < 0.001) and higher European system for cardiac operative risk evaluation (EuroSCORE) (1.5 ± 3.4 vs. 3.3 ± 5.5, P < 0.001). However, after IPTW, no significant differences in demographic data were observed between the groups. Table 2 describes the baseline characteristics of the study patients.

**Table 2:** Baseline characteristics.

	Before IPTW				After IPTW			
Variables	HTK (N = 190)	DN (N = 180)	P value	SMD	HTK (N = 170)	DN (N = 230)	P value	SMD
Age, years	67.8 ± 10.8	68.0 ± 12.0	0.866	0.018	68.5 ± 10.9	67.3 ± 11.5	0.569	0.108
Female, n (%)	85 (44.7)	88 (48.9)	0.487	0.083	80 (47.1)	116 (50.4)	0.746	0.074
Body surface area, m^2^	1.65 ± 0.16	1.64 ± 0.19	0.538	0.064	1.65 ± 0.16	1.66 ± 0.17	0.423	0.095
NYHA class 3–4, n (%)	20 (10.5)	45 (25.0)	<0.001	0.386	25 (14.7)	77 (33.5)	0.096	0.446
Hypertension, n (%)	117 (61.6)	119 (66.1)	0.425	0.094	103 (60.6)	165 (71.7)	0.170	0.241
Diabetes mellitus, n (%)	57 (30.0)	33 (18.3)	0.783	0.042	51 (30.0)	101 (43.9)	0.240	0.287
Stroke, n (%)	27 (14.2)	25 (13.9)	>0.999	0.009	22 (12.9)	25 (10.9)	0.642	0.065
Atrial fibrillation, n (%)	11 (5.8)	10 (5.6)	>0.999	0.010	11 (6.5)	11 (4.8)	0.621	0.064
COPD, n (%)	7 (3.7)	15 (8.3)	0.095	0.197	9 (5.3)	53 (23.0)	0.058	0.522
Dialysis, n (%)	8 (4.2)	5 (2.8)	0.635	0.079	7 (4.1)	6 (2.6)	0.476	0.093
EuroSCORE, %	1.5 ± 3.4	3.3 ± 5.5	<0.001	0.397	1.7 ± 4.1	2.7 ± 4.4	0.053	0.230
Previous OHS, n (%)	13 (6.8)	12 (6.7)	>0.999	0.007	13 (7.6)	17 (7.4)	0.910	0.016
AS, n (%)	134 (70.5)	86 (47.8)	<0.001	0.476	106 (62.4)	153 (66.5)	0.621	0.091
AR, n (%)	32 (16.8)	23 (12.8)	0.341	0.115	30 (17.6)	31 (13.5)	0.450	0.112
ASR, n (%)	20 (10.5)	67 (67.2)	<0.001	0.659	29 (17.1)	40 (17.4)	0.943	0.011
IE, n (%)	4 (2.1)	4 (2.2)	>0.999	0.008	5 (2.9)	5 (2.2)	0.764	0.044
LVEF,%	56.0 ± 10.2	54.9 ± 9.6	0.299	0.108	55.4 ± 10.3	53.7 ± 9.1	0.382	0.175
Hemoglobin, g/dL	12.8 ± 1.9	12.8 ± 1.8	0.731	0.036	12.7 ± 1.9	12.6 ± 1.8	0.839	0.043

PS, propensity score; HTK, Histidine-tryptophan-ketoglutarate; DN, Del Nido; SMD, standardized mean difference; NYHA, new yourk heart association; COPD, chronic obstructive pulmonary disease; OHS, open heart surgery; AS, aortic stenosis; AR, aortic regurgitation; ASR, aortic steno-regurgitation; IE, infective endocarditis; LVEF, left ventricular ejection fraction.

### Operative data

**Fig 1:**
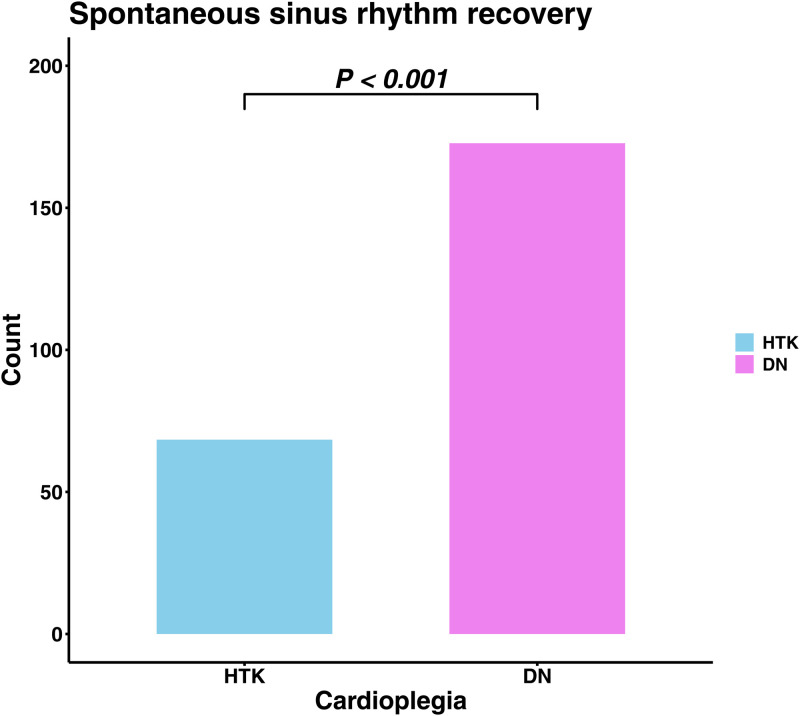
Comparison of the incidence of spontaneous sinus rhythm recovery after aortic cross clamp release between the HTK group and DN group. HTK; Histidine-tryptophan-ketoglutarate; DN, Del Nido.

The operative data was shown in Table 3. The cardiopulmonary bypass time, ACC time and, percentage of minimally invasive cardiac surgery (MICS) did not differ between the two groups after IPTW. The DN group showed higher use of sutureless valve (0.0% vs. 17.4, P = 0.008), and higher rate of spontaneous rhythm recovery without electrical intervention (e.g., defibrillation) after aortic cross clamp release (40.0% vs. 75.2%, P < 0.001, Fig 1).

**Table 3:** Operative data.

	Before IPTW				After IPTW			
Variables	HTK (N = 190)	DN (N = 180)	P value	SMD	HTK (N = 170)	DN (N = 230)	P value	SMD
CPB time, min	134.5 ± 47.4	104.4 ± 35.1	<0.001	0.724	125.5 ± 41.1	157.9 ± 83.0	0.261	0.495
ACC time, min	103.7 ± 35.0	76.0 ± 25.8	<0.001	0.904	95.6 ± 30.5	119.5 ± 64.4	0.298	0.473
MICS, n (%)	64 (3.7)	83 (46.1)	0.020	0.256	49 (28.8)	84 (36.5)	0.375	0.165
Types of prosthesis								
Bioprostheses, n (%)	158 (83.2)	150 (83.3)	>0.999	0.005	148 (87.1)	187 (81.3)	0.316	0.148
Mechanical prostheses, n (%)	32 (16.8)	29 (16.1)	>0.961	0.020	22 (12.9)	42 (18.3)	0.345	0.139
Sutureless valve, n (%)	0 (0.0)	57 (31.7)	<0.001	0.963	0 (0.0)	40 (17.4)	0.008	0.649
Spontaneous rhythm recovery (without electrical intervention)	80 (42.1)	141 (78.3)	<0.001	0.797	68 (40.0)	173 (75.2)	<0.001	0.758

PS, propensity score; HTK, Histidine-tryptophan-ketoglutarate; DN, Del Nido; CPB, cardiopulmonary bypass; ACC, aortic cross clamp; MICS, minimally invasive cardiac surgery.

### Early outcomes

**Fig 2:**
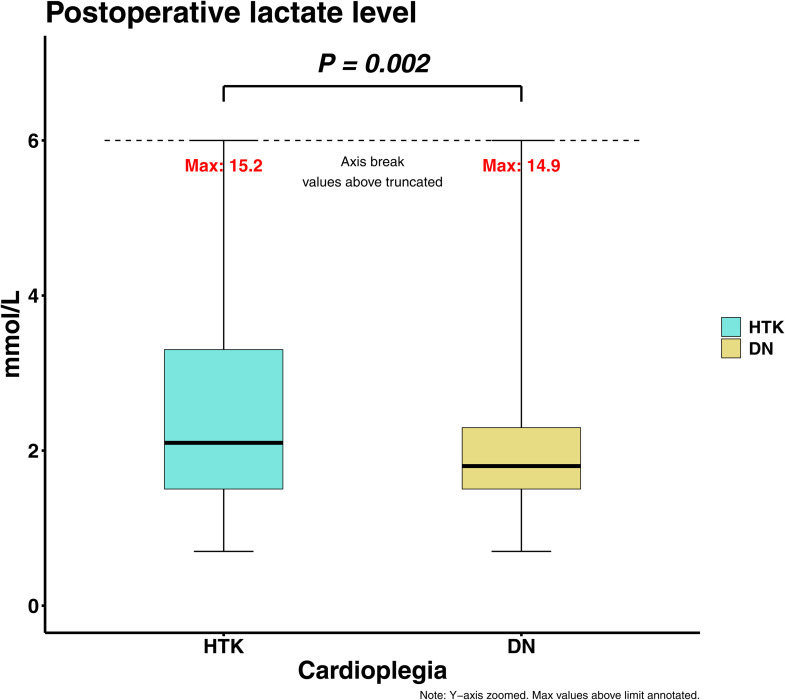
Boxplot for postoperative lactate level of the HTK group and DN group. HTK; Histidine-tryptophan-ketoglutarate; DN, Del Nido.

Early mortality after AVR was not different between the two groups (2 (1.2%) in HTK vs. 2 (0.9%) in DN group, P = 0.554). Postoperative left ventricular ejection fraction (LVEF) did not differ significantly between the two groups. However, the incidence of low cardiac output syndrome (LCOS) was significantly lower in the DN group compared to the HTK group (16 (9.4)% in HTK vs. 4 (1.7%) in DN, P < 0.001). Similarly, the immediate postoperative lactate level was significantly lower in the DN group (3.0 ± 2.6 mmol/L in HTK vs. 2.2 ± 1.4 mmol/L in DN, P = 0.002, Fig 2). Perioperative transfusion requirements and postoperative hemoglobin levels were comparable between the two groups (Table 4).

**Table 4:** Early outcomes.

	Before IPTW				After IPTW			
Variables	HTK (N = 190)	DN (N = 180)	P value	SMD	HTK (N = 170)	DN (N = 230)	P value	SMD
Early mortality, n (%)	2 (1.1)	3 (1.7)	0.951	0.053	2 (1.2)	2 (0.9)	0.554	0.059
Infection, n (%)	16 (8.4)	9 (5.0)	0.270	0.137	13 (7.6)	7 (3.0)	0.065	0.212
Respiratory complications, n (%)	10 (5.3)	6 (3.3)	0.512	0.095	9 (5.3)	5 (2.2)	0.204	0.152
Arrhythmia, n (%)	45 (23.7)	51 (28.5)	0.351	0.110	39 (22.9)	103 (44.8)	0.047	0.475
LCOS, n (%)	16 (8.4)	6 (3.3)	0.065	0.218	16 (9.4)	4 (1.7)	<0.001	0.333
Initial postoperative lactate level, mmol/L	2.9 ± 2.4	2.3 ± 1.8	0.007	0.286	3.0 ± 2.6	2.2 ± 1.4	0.002	0.398
Postoperative LVEF, %	51.9 ± 10.1	51.1 ± 9.3	0.430	0.082	51.3 ± 10.3	53.4 ± 9.3	0.306	0.210
Intraoperative RBC transfusion, pack	1.9 ± 2.2	1.4 ± 1.4	0.006	0.291	1.9 ± 2.0	2.2 ± 2.2	0.722	0.127
Postoperative RBC transfusion, pack	0.4 ± 0.9	0.3 ± 0.9	0.128	0.159	0.4 ± 1.0	0.2 ± 0.8	0.083	0.218
Postoperative hemoglobin level, g/dL	11.4 ± 1.6	11.5 ± 1.2	0.283	0.112	11.3 ± 1.6	11.4 ± 1.1	0.439	0.097
Hospital stay, days	13.4 ± 27.0	11.2 ± 11.7	0.314	0.106	13.4 ± 25.0	10.4 ± 9.2	0.130	0.157

PS, propensity score; HTK, Histidine-tryptophan-ketoglutarate; DN, Del Nido; LCOS, low cardiac output syndrome; LVEF, left ventricular ejection fraction; RBC, red blood cell.

### Late outcomes

**Fig 3:**
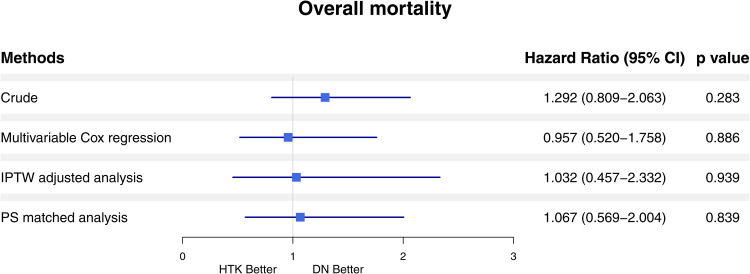
Forest plot for overall mortality: HTK vs. DN Cardioplegia across multiple analytic models. HTK; Histidine-tryptophan-ketoglutarate; DN, Del Nido; IPTW, inverse probability of treatment weighting; PS, propensity-score.

Fig 3 demonstrates that there was no statistically significant difference in overall mortality between the HTK and DN cardioplegia groups across all analytic approaches, including crude comparison, multivariable Cox regression, IPTW-adjusted analysis, and propensity score-matched analysis.

## Discussion

The modified bloodless DN cardioplegia demonstrated favorable early and late outcomes in patients undergoing isolated AVR compared to the HTK solution. The incidence of low cardiac output syndrome and the initial postoperative lactate level were both significantly lower in the bloodless DN group. In addition, the bloodless DN group demonstrated a significantly higher rate of spontaneous restoration of sinus rhythm following aortic cross-clamp removal, further supporting the cardioprotective efficacy of the DN solution. These findings indicate that DN cardioplegia may provide superior myocardial protection and reduce the risk of myocardial injury relative to HTK. Other postoperative complications, including morbidity and mortality, were not significantly different between the two groups.

The comparison between the original DN cardioplegia and HTK solution in myocardial protection has been a topic of increasing interest, particularly as both solutions offer single-dose protection with prolonged ischemic times. Although previous studies suggested similar outcomes of myocardial protection in both the DN and HTK solution, the DN cardioplegia provides several advantages than HTK solution. It requires less volume which offer less hemodilution, higher rate of spontaneous rhythm recovery that suggesting a lower ischemia-reperfusion injury risk, and cost effectiveness [4]. The DN cardioplegia tends to show better early cardiac recovery and less myocardial edema in some studies, while HTK offers strong protection but might be associated with slower recovery postoperatively [20]. In addition, the cost of the DN solution is not expensive compared to HTK solution (1L DN: $ 8 vs 1L HTK: $ 105), it is more affordable for patients in financial difficulty.

Research on modified versions of DN cardioplegia has been expanding, particularly since its patent expired. Studies are investigating various modifications to improve outcomes, especially in adult cardiac surgeries. There are reports of cardiac surgery cases where increasing the proportion of blood was applied. A study reported the outcomes of coronary artery bypass grafting with left ventricular dysfunction patients using modified DN cardioplegia with increased amount of blood rather than original form. They demonstrated advantages such as significant reduction in postoperative epicardial edema, suggesting improved myocardial protection [21]. The approach they used was to add the cardioplegic additives directly to the patient’s whole blood rather than in a crystalloid base. Another study showed the results with the need for defibrillation was found to be significantly less in the modified versions of DN cardioplegia group compared with the classical DN group. There was no statistically significant difference between modified group and classical DN in all parameters related to myocardial protection [22]. There have also been studies published using different base solutions from the original formula. Due to the unavailability of the original ingredients (Plasmalyte A®), they used a modified version of the DN cardioplegia using Ionosteril as base solution. Modified version of DN cardioplegia based on Ionosteril® solution showed equivalent protection compared to Custodiol for isolated mitral valve repair [23].

The effect of blood in the original DN cardioplegia solution is multifaceted and contributes to enhanced myocardial protection during cardiac surgery. It optimizes myocardial protection by supporting oxygen delivery, buffering capacity, nutrient supply, reduced hemodilution, and improved rheology [24]. These are similar to the well-known benefits of blood cardioplegia. However, in DN cardioplegia, blood is mixed with the crystalloid solution in a 1:4 ratio (blood to crystalloid), and there is a lack of scientific evidence that this dose is sufficient to provide the benefits of blood for myocardial protection. Some studies suggested that adding blood to cardioplegia may not provide favorable influences, especially at lower temperatures [25,26]. Dr. Pedro J. DN, who is the pioneer of development of the DN cardioplegia solution, has also commented that the inclusion of blood in the solution may not provide significant additional safety or efficacy in terms of myocardial protection for the short-term myocardial ischemia in pediatric or simpler adult cardiac cases [27]. In our institution, a modified bloodless cardioplegia technique has been utilized since the early 2000s for congenital heart surgeries. After achieving positive results in congenital heart surgery [28], it was subsequently incorporated into adult cardiac surgery in 2014. Our institutional experiences including this study, also advocate the hypotheses that blood components may not be essential for DN cardioplegia. Considering the simplicity and resource efficiency of the operation, eliminating the blood mixing procedure could be a viable option. Bloodless DN cardioplegia eliminates the need for specialized circuits or additional resources to mix blood with the solution, making it an attractive option in resource-constrained settings. In addition, it could be beneficial in particular patients such as, small neonates, infants, or even small adults, for whom incorporating even small amount of the patient’s blood into the cardioplegia can be discouraging. Our study demonstrated that the key benefits traditionally attributed to the original Del Nido cardioplegia were maintained despite the exclusion of blood components from the solution.

Our study has several limitations. First, as a retrospective, non-randomized study conducted at a single institution, it may be subject to selection bias. Although IPTW analysis (with some variables still showing high standardized mean differences) and other statistical methods were employed to mitigate potential biases in patient selection, unidentified confounding factors could still affect the results. Second, data on key cardiac biomarkers, such as troponin and creatine kinase-muscle/brain, were not available, as these markers were not routinely measured following cardiac surgery at our institution except coronary artery bypass grafting. Instead, we included postoperative lactate levels, which may serve as an indirect indicator of postoperative LCOS. Third, in complex or extended cardiac surgeries that multi-dose administration of cardioplegia is necessary due to prolonged ACC time, it would be valuable to investigate whether the use of bloodless DN cardioplegia yields a distinct impact compared to other cardioplegia strategies. To validate our findings, multi-center, randomized controlled trials with a larger patient cohort would be required to better assess the myocardial impact of the modified bloodless DN cardioplegia compared to original formula.

## Supporting information

S1 Fig Love Plot demonstrating covariate balance before and after IPTW adjustment. (PDF)

S2 Fig Density Plot of propensity score distributions showing overlap between the HTK and DN groups. (PDF)
