# Supplementary figures and images for: The efficacy of modified bloodless del Nido cardioplegia in isolated aortic valve replacement
Source: PLoS One. 2025 Sep 29;20(9):e0333083. doi: 10.1371/journal.pone.0333083 (PMC12478951; doi:10.1371/journal.pone.0333083)

# Covariate Balance

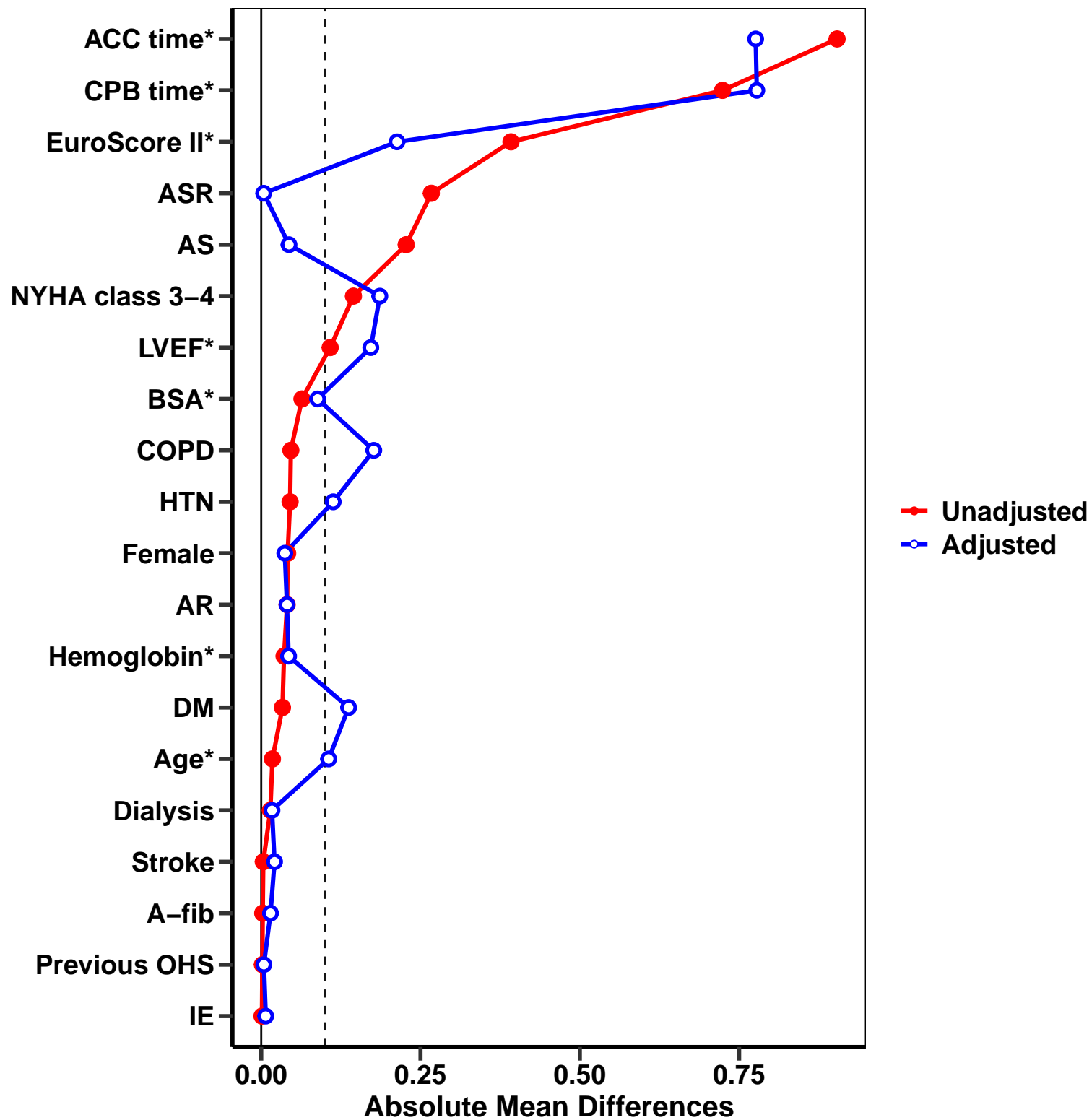

Supplement: S1 Fig — (PDF) [file pone.0333083.s001.pdf]

**Before adjustment**

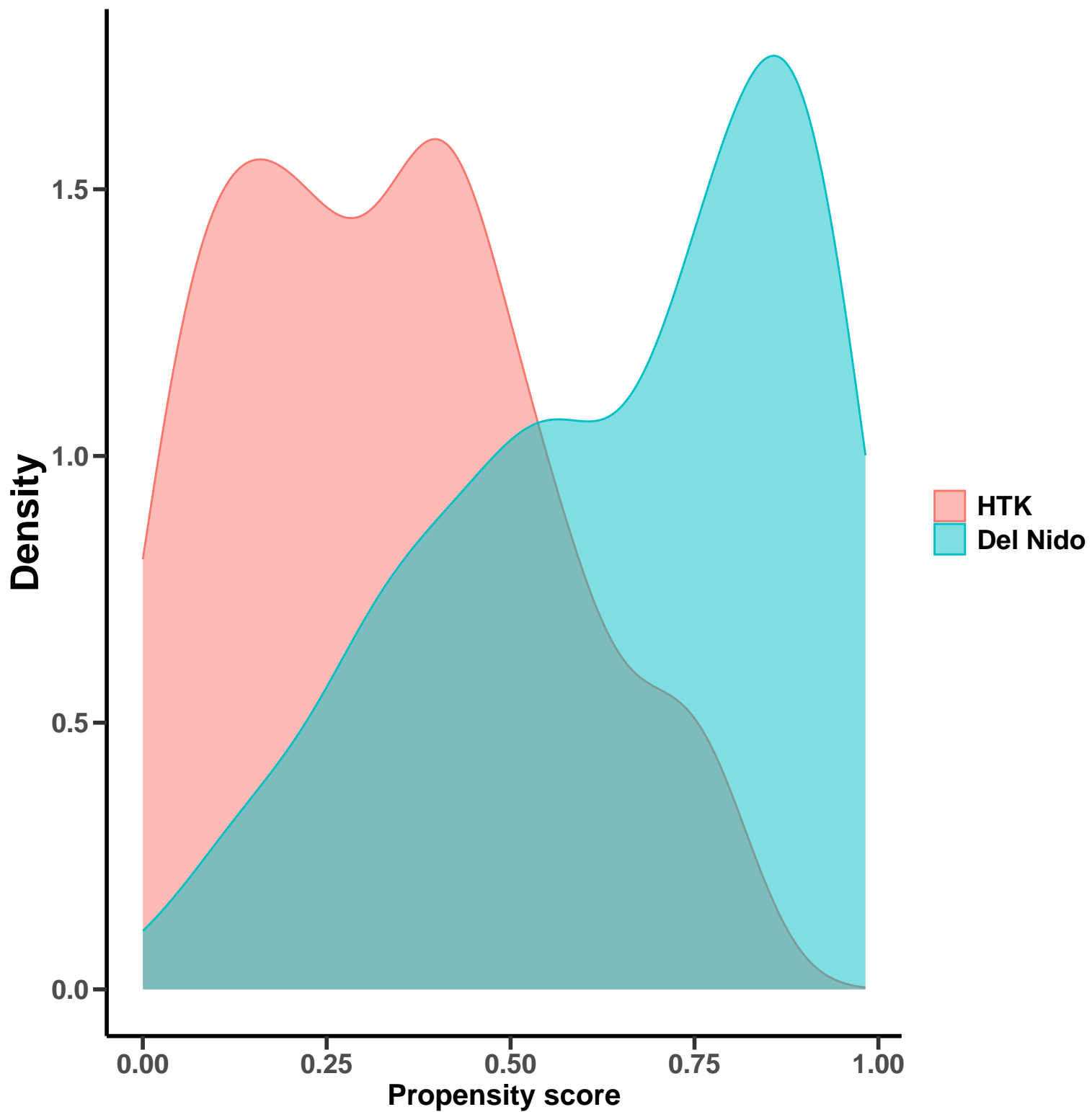

**After IPTW**

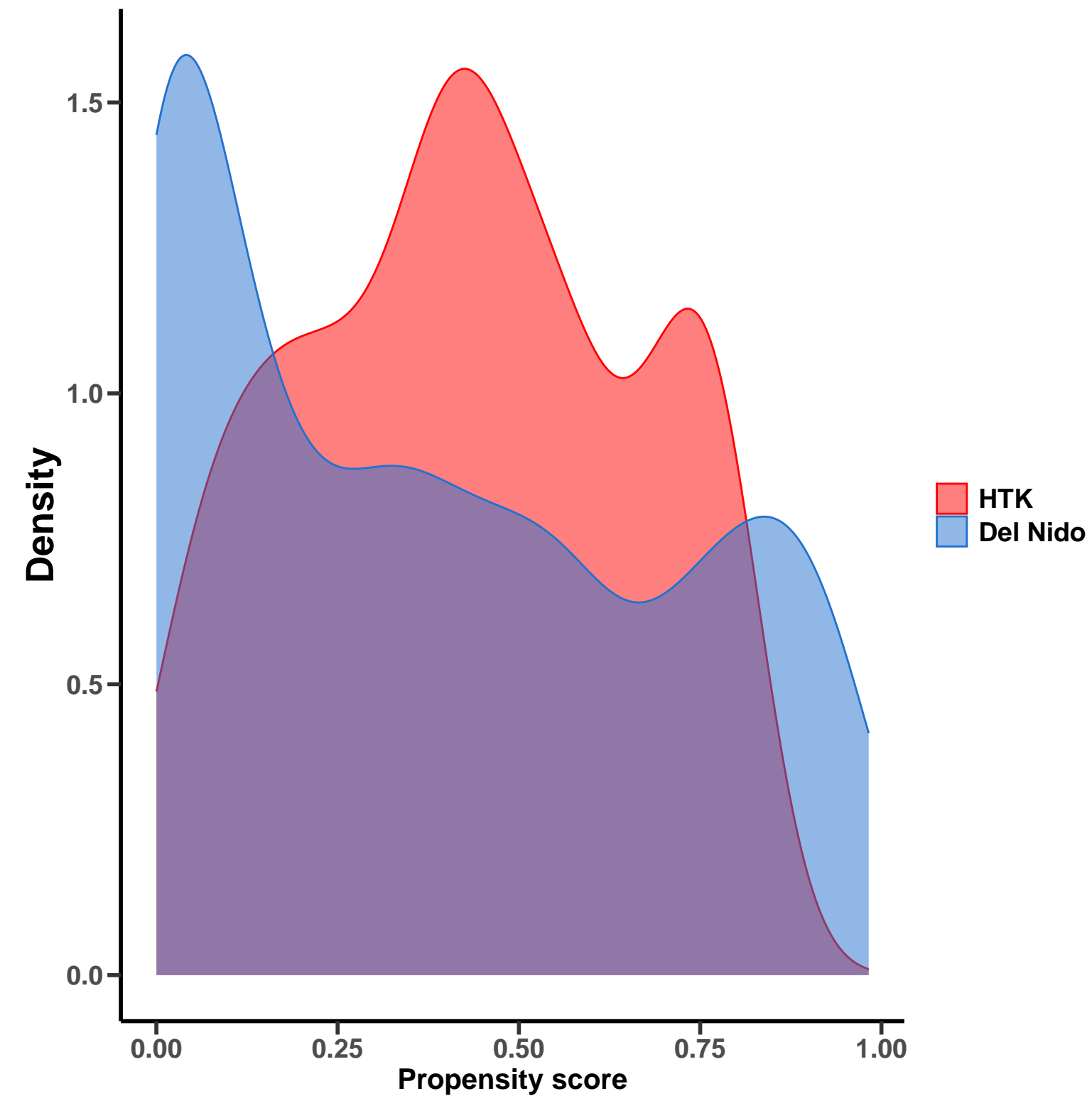

Supplement: S2 Fig — (PDF) [file pone.0333083.s002.pdf]
